# Interaction Analysis through Proteomic Phage Display

**DOI:** 10.1155/2014/176172

**Published:** 2014-09-11

**Authors:** Gustav N. Sundell, Ylva Ivarsson

**Affiliations:** Department of Chemistry-BMC, Uppsala University, P.O. Box 576, 751 23 Uppsala, Sweden

## Abstract

Phage display is a powerful technique for profiling specificities of peptide binding domains. The method is suited for the identification of high-affinity ligands with inhibitor potential when using highly diverse combinatorial peptide phage libraries. Such experiments further provide consensus motifs for genome-wide scanning of ligands of potential biological relevance. A complementary but considerably less explored approach is to display expression products of genomic DNA, cDNA, open reading frames (ORFs), or oligonucleotide libraries designed to encode defined regions of a target proteome on phage particles. One of the main applications of such proteomic libraries has been the elucidation of antibody epitopes. This review is focused on the use of proteomic phage display to uncover protein-protein interactions of potential relevance for cellular function. The method is particularly suited for the discovery of interactions between peptide binding domains and their targets. We discuss the largely unexplored potential of this method in the discovery of domain-motif interactions of potential biological relevance.

## 1. Introduction

The human interactome is estimated to contain about 130,000 binary protein-protein interactions (PPIs), of which the majority remains to be discovered [1]. PPIs are crucial for cellular function and dysfunction and large efforts are therefore invested in their identification and in constructing PPI based networks [2]. Different high-throughput methods render complementary data. For example, affinity purification coupled to mass spectrometry (AP-MS) [3, 4] and luminescence-based mammalian interactome mapping (LUMIER) [5] provide information on complexes, and yeast-two-hybrid (Y2H) experiments give insights into binary PPIs [1], as summarized in Table 1. Despite the significant advances being made the last decade, the human interactome is still largely uncharted and the accumulated knowledge is biased towards well-studied proteins [1, 6].

Particularly elusive to high-throughput methods are the interactions between peptide binding domains and their target motifs, which are typically less than ten residues in length [7, 8]. The peptide motifs are typically located in regions of intrinsic disorder, which can be found in about 35% of the human proteins [9]. Currently, there are more than 2,400 instances reported in the eukaryotic linear motif (ELM) resource for functional sites in proteins [10], including binding motifs and posttranslational modification sites. This, however, covers only a fraction of the motifs expected to be present in the human proteome [8].

Among the most abundant peptide binding domains in the human proteome are the PSD-95/Discs-large/ZO-1 (PDZ) domains that typically interact with C-terminal sequences of target proteins [11]. Other domains, such as the Src Homology 2 (SH2), bind to phosphorylated target motifs [12]. Domain-motif interactions tend to be of rather low affinities and hence are easily lost in methods such as AP-MS. Although difficult to capture experimentally, transient protein-peptide interactions are crucial for cell function and may be perturbed by disease-causing genetic variations or by viral interferences [13, 14].

Phage display is a powerful approach for establishing binding preferences of peptide binding domains and in extension to discover novel motifs. In combinatorial peptide phage display, highly diverse libraries are used to identify high-affinity ligands with potential to serve as inhibitor [15]. Consensus motifs are derived based on the retained sequences and can be used for predictions of potential ligands in a target proteome [16]. These predictions, however, are not always accurate, which can lead to tedious experimental validations of putative targets. Luck and Travé demonstrated that predictions of human PDZ domain ligands based on results of combinatorial phage display may be hampered due to a bias towards overly hydrophobic (i.e., Trp containing) peptides [17].

**Figure 1:**
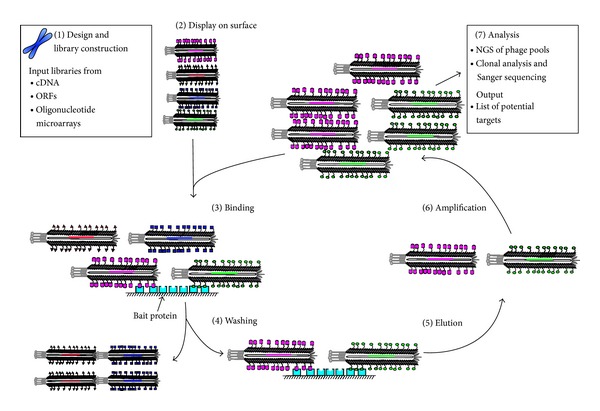
Schematic representation of proteomic phage display using the M13 phage. Input phage display libraries are constructed from cDNA, ORFs, or oligonucleotide arrays designed from a proteome of interest (1). Peptides are displayed on pVIII (2). Bait proteins are immobilized on a solid surface and incubated with the naïve input phage library (3). Binding of phage occurs through interactions between displayed peptides and bait proteins, but nonspecific interactions cause noise in the selection (not shown). Unbound phage is washed away (4) and bound phage is eluted through acidic or basic conditions or by the addition of actively growing host bacteria (5). Eluted phage is amplified (6) and used for repeated (typically 3–5) cycles of selection, which is necessary to amplify specifically bound phage over nonspecific binders. Sanger sequencing of confirmed binders and/or NGS of the retained phage pools provides lists of binders from the target proteome (7).

A promising strategy to discover novel protein-motif interactions is to reduce the search space to comprise only sequences of a target proteome. In such proteomic phage display, expression products from genomic DNA, cDNA, open reading frames (ORFs), or from designed synthetic oligonucleotides are displayed on phage particles (Figure 1). Proteomic phage display has been used for the identification of allergens [18], antibody epitopes, tumor polypeptides producing immune response [19], and PPIs as well as for the identification of proteins binding to phospholipids and small chemical compounds [20, 21]. In this review, we survey the features, the development, and the applications of various phage display systems used for proteomic phage display, with a particular focus on the elucidation of cellular PPIs. For extensive reviews on cDNA/ORF display for antibody epitope mapping of antigen and pathogen research we refer the readers to dedicated reviews [22–26].

## 2. Phage Display Systems Used for Proteomic Phage Display

**Figure 2:**
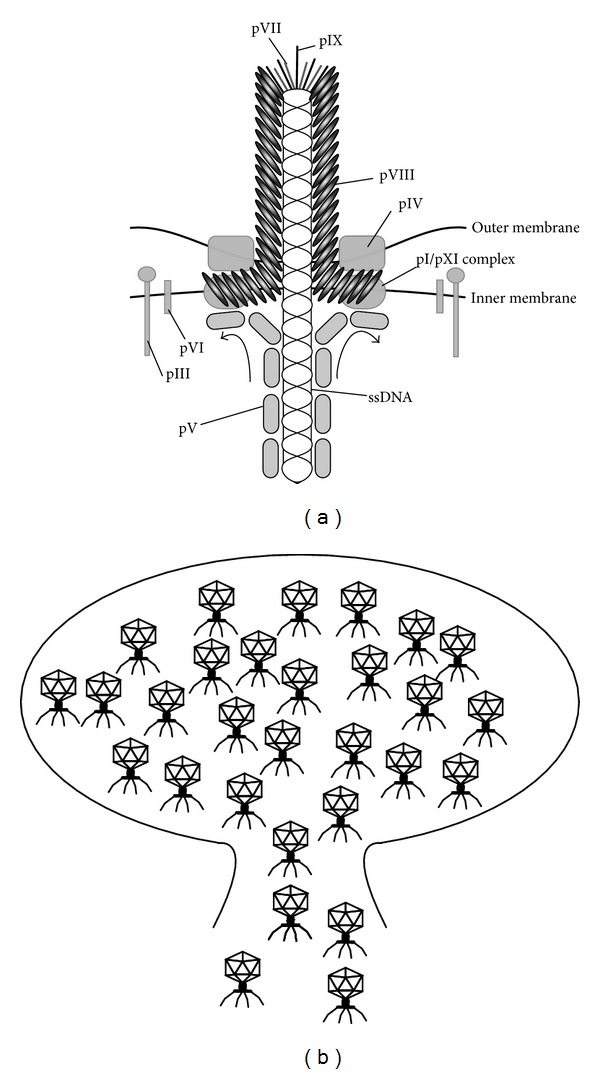
Schematic representation of the assembly and excretion of the M13 filamentous phages and the exit through cell lysis of the lytic T7 phage. (a) The filamentous M13 phage is assembled at the cell membrane of the bacterial host. In the host cell, the ssDNA is protected by association with protein pV, which detaches at the membrane upon assembly. At the start of the assembly, membrane associated pVII/pIX bind a specific DNA packing signal. Membrane bound protein pVIII binds to the DNA and is transferred across the membranes. The transport is facilitated by a complex of pI and pXI situated in the inner membrane and protein pIV that makes a pore through the outer membrane of the bacteria for the phage to pass through. As a final step, pVI and pIII that span the inner membrane are assembled on the phage. The figure was created based on [31]. (b) The lytic T7 phage, schematically shown with its typical icosahedral head, is assembled in the cytosol of the host cell. It is multiplied to such extent that the host cell finally bursts and the phage is released to the surrounding.

Phages that have been used for proteomic phage display include the filamentous M13 phage, the lytic T7 phage, and the temperate phage *λ*. The main advantage of the M13 phage display system is the ease of its manipulation and handling as detailed in the following section. The main drawback of the M13 phage is that the displayed proteins are secreted through the periplasmic space of the* Escherichia coli* membrane (Figure 2(a)), which may confer problems with unpaired cysteine residues that form undesired disulfide bridges and thereby compromise the successful display of designed sequences [27]. The T7 phage and the *λ* phage do not suffer from this issue, as they are not passing through the membrane but lysing the host cells (Figure 2(b)).

### 2.1. M13 Filamentous Phage

The M13 filamentous bacteriophage was the first developed phage display system [28]. The M13 phage consists of a circular single stranded DNA (ssDNA) that is covered by five different coat proteins (pIII, pVI, pVII, pVIII, and pIX). The 2,700 copies of the major coat protein pVIII cover the length of the phage [29]. The minor coat proteins pVII and pIX cover one end of the phage particle and pIII and pVI the other end (Figure 2(a)). The minor coat protein pIII is crucial for infection as it initiates the interaction with the F-pilus and TolA receptor [30]. For details on the structure and assembly of filamentous bacteriophages we refer the reader to an extensive review on the topic [31]. Typically, phage propagation is uncoupled from expression and display of desired peptide on the phage particle. This is accomplished through hybrid systems where a phagemid is used for library construction and helper phage is added to provide the information needed for assembly of the phage particle [32].

The M13 phage is a highly versatile system as distinct coat proteins can be used for N- or C-terminal display and for monovalent or multivalent display, respectively [33]. Commonly, the pIII protein is used for low valency display (one to five copies per phage) and the pVIII for high valency display, with up to 1,000 copies per phage in evolved hybrid systems [23]. Highly diverse M13 phage libraries (up to 10^10^) can be constructed due to the fact that M13 has a circular ssDNA. For a typical library construction, an oligonucleotide library is designed complementary to the ssDNA with flanking regions corresponding to the phagemid vector. The oligonucleotides are then annealed to the vector and the complementary strand is synthesized and ligated together to form a circular, double stranded DNA vector, which is then electroporated into* E. coli* [34].

### 2.2. T7 Phage

T7 phage is an icosahedral virus of the Podoviridae family and has a linear double stranded (ds) DNA genome. In contrast to M13, T7 is not secreted but released from the host cell through lysis (Figure 2(b)). The T7 phage starts to reproduce immediately upon infection, which is continuous until the point of cell lysis. The major capsid protein (gp10) is encoded by gene 10 and makes up about 90% of the icosahedron capsid. This gene yields two products, 10A and 10B, in a nine-to-one ratio. The minor protein 10B results from a frame shift in the end of the gene that makes the capsid protein 52 residues longer [35]. Fusion proteins are displayed on protein 10B C-terminally of the 52 extra residues. Depending on the system used, up to 1,200 amino acid inserts can be displayed at low valency (5–15 copies per virion) or shorter inserts (up to 50 amino acids) at higher valency (up to 415 copies) [36]. The linear genome makes it more challenging to construct T7 phage libraries as compared to M13 libraries. Library construction includes two-step ligations and the* in vitro* packing of DNA into the phage, which in the T7 select system (Novagen) is accomplished by the addition of DNA to commercially available packaging extract. The packaging extract is sensitive to work with and rather costly if larger libraries are prepared [33].

### 2.3. Lambda Phage

The temperate *λ* phage has an icosahedral head. The main structure of the shell is built from the major coat protein gpE (415 copies) and is stabilized by the capsid protein gpD (402–420 copies) [37]. The head is linked to a flexible helical tail constructed by disks of the major tail protein gpV. Its linear dsDNA is packed in the bacteriophage head. The DNA is injected into the host bacteria and is stably integrated into the host chromosome during the lysogenic state. When triggered correctly, the *λ* phage starts a lytic cycle [38].

Both the tail protein gpV and the head protein gpD have been used for phage display. Initially, the *λ*foo vector was constructed for the C-terminal display on gpV, with a low display level that made it suitable for capturing high-affinity interactions [39]. Later, systems were developed for the display of peptides N-terminally or C-terminally to the major coat protein gpD [40–42]. Libraries with diversities in the range of 10^7^–10^8^ are constructed using commercially available* in vitro* packaging systems.

## 3. Proteomic Phage Display

Over the years, different approaches towards proteomic phage display have been taken, from cDNA and ORF display to the display of the expression products of highly defined synthetic oligonucleotide libraries, as detailed in the following section.

### 3.1. cDNA/ORF Display

In cDNA display, a gene, a cDNA, or a complete genome is displayed on phage particles. Theoretically, this is a straightforward technique. However, it suffers from difficulties in obtaining high-quality libraries [43]. This is a consequence of the transcriptional stop codons at the 3′-end of coding regions, the polyA tail of mRNA, and the often nondirectional cloning. The fraction of clones expressing peptides in frame in a naïve cDNA library may be as low as 6%. Additionally, phage with truncated constructs tends to outgrow clones with correctly displayed sequences [44, 45]. The quality can be improved by using ORF enriched DNA collections for library construction [43, 46]. Library quality can further be improved by fragmentation of the DNA by, for example, treatment with Deoxyribonuclease I or by sonication before cloning [26, 47]. Despite the quality issues, a variety of libraries based on human or pathogen cDNA/ORFs have been derived and used for PPI screening.

#### 3.1.1. cDNA/ORF Display Using the M13 Filamentous Phage

A number of studies have employed the M13 filamentous phage system for cDNA display. In most cases, the expression products are displayed on the pIII protein either indirectly through the Jun-Fos system or directly [48]. There are also reports of N-terminal multivalent display on pVIII [49] and monovalent C-terminal display on pVI [50]. When displaying inserts N-terminally, a main limitation is that the inserts have to be in the same reading frame as the pIII or pVIII proteins and that there can be no in frame stop codons. A way to enrich for ORFs and the correct presentation of encoded sequences and thereby improve the quality is the so-called Hyperphage [51]. In this system, the helper phage has a truncated g3 so that the phagemid pIII fusion is the only source of pIII, as originally described by Kristensen and Winter [52]. This strategy has been used for the successful identification of immunogenic polypeptides of* Mycoplasma hyopneumoniae* [53]. Recently, a novel trypsin-sensitive helper phage was derived for a similar purpose [54].

*Indirect Display on pIII: The Jun-Fos System.* A system for indirect cDNA display on pIII termed pJun-Fos was engineered in 1993. This system takes advantage of the strong association between the Leucine Zipper Jun and Fos [55]. The pIII-fused Jun and Fos-linked cDNA expression product is assembled in the* E. coli* periplasm, which leads to the indirect display of the functional expression products of cDNA on pIII. The complex is stabilized by disulfide bridges between cysteines engineered at the N- and C-termini of Jun and Fos. In the original publication, enzymatically active alkaline phosphatase was displayed on pIII and the authors discussed the potential of the system as a tool in PPI screening. The Jun-Fos system has since then been a popular cDNA display system for the discovery of antibody epitopes [56] as reviewed elsewhere [48].

A Jun-Fos system modified to ensure cloning in all three reading frames was used to identify host-pathogen protein-protein interactions between the ribonucleoproteins of influenza virus and the expression products of a human cDNA library (inserts >750 bp) [57]. In this study, the authors pinpointed a direct interaction between the A domain of human high mobility group box proteins and the viral bait protein.

*Direct Display on pIII. *Expression products of cDNA/ORF have also been displayed directly on pIII. In an early study, the plasminogen-activator inhibitor 1 was fragmented into 50–200 bp and cloned into the M13 gpIII phagemid vector [58]. This library was used for epitope mapping of a monoclonal antibody raised against this protein. For PPI screening, Hertveldt et al. constructed a phage library by fusing genomic* S. cerevisiae* DNA (100–1,500 bp) to gpIII lacking the N1 domain [59]. From panning of the yeast cDNA library against GAL80, fragments of the known binder GAL4 and three other ligands of potential physiological relevance were retrieved, thereby demonstrating that the system can be used for identification of biologically relevant targets. Around the same time, Yano et al. constructed a fragmented genomic* E. coli* pIII library and identified binders to alkaline phosphatase [60].

Two other studies demonstrate that ORF enriched cDNA display on pIII can be used to identify targets of potential biological relevance. In the first study, the interactomes of the high mobility group A proteins HMGA1 and HMGA2 were elucidated using an ORF enriched murine cDNA M13 pIII library displaying 200–500 base pair fragments [61]. For these nuclear chromatin factors, four targets were identified, namely, TBP associated factor 3b and chromatin assembly factor I, subunit A, and two previously uncharacterized proteins. For the first two proteins, interactions were confirmed between the full-length proteins through GST-pull down assays and coaffinity purification of overexpressed proteins in HEK293T cells [61].

In the second study, an ORF enriched and fragmented cDNA library displayed on pIII was used for interactome mapping of transglutaminase 2 (TG2) [62]. Through next-generation sequencing (NGS) of selected phage pools a list of potential targets was retrieved. The most frequently occurring ligands interactions were validated through protein complementarity assays with 80% success rate, thus demonstrating the power of the combination of ORF enriched cDNA display and NSG in interactome mapping.

*Posttranslational Modifications and ORF Display on pIII. *PPIs are often controlled by posttranslational modifications, with the most common modifications in eukaryotic proteomes being phosphorylation of Ser/Thr/Tyr residues [63]. These modifications can create or abrogate binding sites or modulate function by more indirect means. A few attempts have been made towards investigating PPIs relying on posttranslational modifications through proteomic phage display. In particular, Cochrane and coworkers used the fyn tyrosine kinase to* in vitro* phosphorylate a fragmented leukocyte cDNA library (10^8^) displayed on pIII. The phosphorylated library was used in selections against the phosphotyrosine binding tandem of SH2 domains of SHP-2 [64]. Nonspecific binding clones were removed before* in vitro* phosphorylation and selection using SHP-2 Sepharose. Through competitive ELISA experiments using phosphorylated phage and synthetic peptides, double phosphorylated PECAM-1 was identified and confirmed as a SHP-2 ligand. It thus appears possible to identify natural interactions relying on posttranslational modification through cDNA phage display. However, given the lack of follow-up studies it does not seem like a feasible way to go for high-throughput analysis of PPIs depending on posttranslational modification. Other attempts to tackle posttranslational modification involved the system for the production and enrichment of phage displaying N-glycoproteins [65].

*cDNA Display on pVI. *A limited set of studies has employed C-terminal cDNA display on pVI, thus circumventing issues related to the presence of premature stop codons. Using a pVI cDNA library of the hookworm* Ancylostoma caninum* ligands were identified for two serine proteases [50]. A few years later, a rat liver cDNA library fused to pVI was used for the identification of peroxisomal proteins by panning the library against antibodies raised against peroxisomal subfractions [66]. In another study, a pVI cDNA library from the colorectal cancer cell line HT-29 was used to identify a panel of candidate tumor antigens [67]. Other studies have reported the discovery of autoantigens for diseases such as multiple sclerosis [68] and rheumatoid arthritis [69]. However, at this stage there are no studies that have applied pVI cDNA display for the explicit purpose of interactome analysis. The monovalent display on pVI makes it less suited for capturing low-affinity interactions.

#### 3.1.2. T7 Phage Display

T7 phage display has become a popular system for cDNA/ORF display, starting from the identification of RNA binding proteins from cDNA displayed on the C-terminus of the capsid protein 10B [70]. It has typically been used for antigen discovery [71]. For example, T7 cDNA display of sea snake venom gland mRNA identified rabbit anti-sea snake venom IgGs as well as new toxins [72]. T7 cDNA display has also been used to explore interactions between parasite proteins and host enterocytes [73, 74].

High-quality ORF T7 display libraries have been used for interactome analysis. In particular, Caberoy and coworkers created a library by combining dual phage display with specific elution of bound phage by protease cleavage [75]. In this system a biotin tag is expressed C-terminally of the inserts and thus is only present when the inserts are in frame. The tag is biotinylated by the* E. coli* BirA enzyme, which enables the selection of ORF clones using immobilized streptavidin. Bound phage is eluted by cleavage with 3C protease. Following this approach, novel tubby binding proteins were identified and then validated through complementary approaches. Of 14 potential target proteins tested, 10 were confirmed as ligands by Y2H and/or pull down assays [75]. The same group used their T7 high-quality ORF library to identify tubby and tubby-like protein 1 as eat-me signals stimulating phagocytosis [76] as well as substrates for the protease calpain 2 [77].

A final example is provided by a study on the suppressor of cytokine signaling 3 (SOC3) [78]. A potential ligand of SOC3, an 11-mer C-terminal peptide of the very long chain acyl-CoA dehydrogenase (VLAD), was found through selections against a human liver cDNA T7 phage library. The interaction was confirmed* in vitro* and in cell-based experiments and was further validated in animal experiments. Based on the results, the authors proposed that SOC3 is an important factor for lipid metabolism.

#### 3.1.3. Phage *λ* cDNA/ORF Display

Phage *λ* cDNA/ORF display has found use in antigen discovery, as reviewed elsewhere [79]. Already in 1997, the *λ*foo system was used for epitope mapping of human galectin [80]. In this study, a library was constructed from fragmented cDNA of galectin-3 and screened against immobilized monoclonal antibodies, leading to the identification of two distinct epitopes of nine and eleven amino-acid residues. This method was shown to outperform a random peptide phage library. Other studies report on epitope mapping of monoclonal and polyclonal antibodies with cDNA phage *λ* libraries from human brain and mouse embryo [41, 81, 82]. However, to our knowledge there are at this stage no papers on interactome analysis using phage *λ*.

### 3.2. Proteomic Peptide Phage Display Libraries from Oligonucleotide Array

Recently, the advances in oligonucleotide microarray synthesis [83] in combination with bioinformatics and NGS have opened new avenues for the construction of highly defined phage libraries. The pioneering study published in 2011 by Larman and coworkers reported the creation of a T7 library displaying 36-mer peptides representing the complete human proteome, with seven amino acids overlaps [84]. With this library, the authors developed a phage immunoprecipitation sequencing platform for the discovery of autoantibodies. They also demonstrated a more general use for interactome mapping by identifying targets for thereplication protein A2.

In a recent study, a previously engineered pVIII phagemid for multivalent C-terminal display [85] was used to create two distinct proteomic peptide phage display (ProP-PD) libraries. The first library was designed to contain all human C-terminal 7-mer peptides whereas the second library contained all C-termini of known viral proteins. After confirmation of composition and coverage of the libraries through NGS they were used in selection against nine PDZ domains of densin-180, DLG1, erbin, and scribble. Phage pools retained after different selection rounds were analyzed through NGS, which provided detailed information on the progress of the selections. Between two and thirty ligands were obtained for each PDZ domain after the fifth round of selection. Of these, more than 50% of the ligands retained for DLG1, densin-180, and erbin were previously known targets. In contrast, only 13% of the scribble ligands were known since previously. Interactions between full-length scribble and the novel ligands plakophilin-4, mitogen-activated protein kinase 12, and guanylate cyclase soluble subunit alpha-2 were confirmed through colocalizations and coimmunoprecipitations, suggesting that ProP-PD identified biologically relevant targets and that the approach can be used to complement PPI networks. The ligands retrieved from the selections against the library designed from virus proteins were mostly established biologically relevant ligands, thus demonstrating that the approach can efficiently identify host-pathogen PPIs of biological relevance. Taken together, the proteomic peptide phage display appears to be a highly useful tool for proteome wide screening of domain-motif interactions.

## 4. Concluding Remarks and Further Perspectives

Various systems for proteomic phage display have been evolved over the last 20 years, with different approaches taken to improve the quality of the displayed sequences. The preferred systems have been the filamentous M13 and the lytic T7 system. The displayed regions range from 7 to 1,500 bps, thus allowing the proteomic identification of peptide ligands as well as interactions involving folded domains. Although most studies have focused on mapping antibody epitopes, it appears as if cDNA/ORF phage display has the potential to successfully identify PPIs of putative biological relevance. This is evident from the validation range of 50–80%, which is considerably better than for techniques such as Y2H [86]. However, cDNA/ORF phage display has had limited use as a method for interactome analysis. The main issue of cDNA/ORF phage display is the lack of control over the displayed sequences, which affects library quality and likely results in the display of a high percentage of unfolded/misfolded proteins and of stretches that are typically inside of folded proteins and not normally available for binding. In most cases, there is a lack of information on the library quality and the coverage of the target genome. Despite the advent of NGS we did not find any publication reporting on the complete sequencing of a cDNA/ORF library, which would provide valuable insights into the quality of the libraries and a better understanding of the interaction space covered during the experiments.

Proteomic peptide phage libraries, created using a combination of bioinformatics and synthetic oligonucleotide libraries, and analyzed through NGS, offer the advantage of full control of displayed regions [87]. At this stage, the cost of highly diverse high-quality oligonucleotide libraries is still rather high. However, given the rapid advances in large-scale* de novo* DNA synthesis [83] we foresee that the cost will go down and that this approach will become increasingly popular for proteomic screening of domain-motif interactions. This will be particularly feasible as phage display can be scaled to hundreds of proteins in parallel [88] and can be paired with NGS of the naïve phage libraries [89] as well as the selected pools, thus providing comprehensive information on the library composition as well as on the retained targets.

By performing proteomic phage display in parallel with other high-throughput methods such as AP-MS or Y2H it is possible to enrich PPI networks with additional interactions and insights on the domain-motif level. Such attempts have previously been made using combinatorial peptide phage display, with an excellent example provided by the Tong et al. study that elucidated the yeast SH3 interactome [90]. More recently, the binding specificities of the worm* Caenorhabditis elegans* SH3 domains were elucidated via high-throughput peptide phage display. The results were combined with the SH3 interactome that was mapped through Y2H experiment [91]. The use of proteomic libraries rather than combinatorial phage libraries for this kind of analysis will obviate the need for predictions as it directly identifies the target protein based on the selected ligands. ProP-PD will be particularly useful in providing unbiased information on domain-motif interactions. This will give novel insights into the function of unexplored motifs in the human proteome. As these experiments can be performed in high-throughput the limiting factor for elucidating domain-motif interactions will be the access to recombinant proteins and the downstream cell biological validations.

## Figures and Tables

**Table 1:** Summary of high-throughput methods for identification of PPIs, types of interactions identified, and major advantages and disadvantages of the respective method.

Method	Type of interaction	Advantage	Disadvantage
AP-MS	Binary and complexes	Physiological	Bias towards stable interactions, limited to specific condition (e.g., cell type)
LUMIER	Binary and complexes	Physiological	Bias towards stable interactions
Y2H	Binary	Low-tech	Bias towards stable interactions, bias towards soluble proteins that can translocate to the nucleus
Combinatorial peptide phage display	Binary	Large library size (up to 10^10^) Identification of consensus motifs	Need for bioinformatics, limited to natural amino acids, limited to protein-peptide interactions
Proteomic phage display	Binary	Identification of target proteins and consensus motif	Limited to natural amino acids
